# A Narrative Review of Current Understanding of the Pathophysiology of Polycystic Ovary Syndrome: Focus on Plausible Relevance of Vitamin D

**DOI:** 10.3390/ijms22094905

**Published:** 2021-05-05

**Authors:** Rajeshwari Kalyanaraman, Lubna Pal

**Affiliations:** 1 Department of Obstetrics and Gynecology, St Francis Hospital and Medical Center, 114 Woodland Street, Hartford, CT 06105, USA; 2 Department of Obstetrics, Gynecology & Reproductive Sciences, Yale School of Medicine, 333 Cedar Street, New Haven, CT 06510, USA; lubna.pal@yale.edu

**Keywords:** polycystic ovarian syndrome, vitamin D, anti-mullerian hormone, vascular endothelial growth factor

## Abstract

Polycystic ovarian syndrome (PCOS) is the most prevalent endocrinopathy of reproductive years. Salient features in presentation of patients PCOS include menstrual dysfunction, hyperandrogenism and/or polycystic appearance of ovaries on ultrasound. While the diagnosis of PCOS depends on presence of specified criteria, misdiagnoses are common. Despite years of extensive research, the exact aetiology of PCOS remains largely unknown. In the past decade, apart from insulin resistance and hyperandrogenemia, anti-mullerian hormone (AMH), an important marker of ovarian reserve, and vascular endothelial growth factor (VEGF), a crucial factor in angiogenesis, have been examined as plausible players of causative relevance for PCOS. Vitamin D, a sex-steroid hormone that is universally known for its relevance for skeletal health, has received increasing attention due to growing evidence supporting its pivotal in reproductive physiology and in PCOS. In this review we summarize our current understanding of the mechanisms relevant to the pathophysiology of PCOS and examine the role of vitamin D signalling in this context.

## 1. Introduction

Polycystic ovarian syndrome (PCOS) is the most commonly diagnosed endocrine disorder amongst reproductive age women, and the leading cause of anovulatory infertility [1]. The prevalence of PCOS ranges between 6% to 10% depending on the diagnostic criteria employed [2]. As discussed elsewhere in this volume, heterogeneity in clinical presentation of PCOS is well recognized, with overt symptoms that range from menstrual dysfunction (spectrum ranging from menses being infrequent, to too frequent or even absent), to features of hyperandrogenism (hirsutism, acne and even alopecia). Polycystic appearing ovaries on pelvic ultrasound and elevated circulating levels of androgens represent “covert” features that may or may not be accompanied by the abovementioned overt clinical stigmata [1,2,3].

Our collective ability to “diagnose” PCOS has been facilitated over recent decades by the emergence of the prevalent diagnostic criteria (Table 1) [2]. Since the original documentation and description by Stein and Leventhal [4], much has been learnt about the spectrum of its symptomatology, its endocrinology, the accompanying metabolic havoc, the potential for co-morbidities and long-term risks linked with PCOS. Negative consequences of this diagnosis affect not just the reproductive wellbeing, but also have wide ranging implications for general as well as long term health of those affected [5,6]. In addition to the classic symptom burden, issues of adiposity, obesity, of mental health, sleep and sexuality are overrepresented in women with PCOS [7,8]. A diagnosis of PCOS has been associated with an enhanced lifetime risk of type 2 diabetes mellitus, sleep apnoea, metabolic syndrome, cardiovascular disease and endometrial cancer [9,10,11,12].

## 2. Pathophysiology of PCOS—Current Understanding Based on the Known and the Hypothesized

Despite years of extensive research, the exact aetiology of PCOS remains largely unknown. However, efforts over the decades have unravelled a complex and critical interplay of neural, endocrine and genetic underpinnings. The ovary, the hypothalamus and genetics have each been hypothesized, systematically examined and largely rejected as the principal instigators. More recently, insulin resistance and hyperandrogenemia have come under the spotlight as key players in the pathogenesis of this complex disorder [13,14,15,16]; insulin sensitizing agents have attained a central place in PCOS management. Metformin is the prototype of insulin sensitizing drugs that is commonly utilized in PCOS management although accruing data in recent years provide reassuring evidence of therapeutic efficacy as well as safety of isomers of inositol (carbocyclic polyols) in improving insulin sensitivity in PCOS populations [17]. Insulin is an important regulator of glucose and lipid metabolism [18]. Hyperandrogenemia is also a known consequence to hepatic and systemic insulin resistance. Hyperinsulinemia resulting from insulin resistance inhibits the hepatic synthesis of sex hormone binding globulin (SHBG) thereby resulting in excess of circulating free androgens [19]. Yet another mechanism whereby insulin resistance and hyperinsulinemia contribute to elevated circulating androgens is through direct stimulatory effects on the ovarian theca [20] and on increasing ovarian androgen production by enhancing responsivity of the theca cells to the luteinizing hormone (LH) [21]. Hyperandrogenemia itself feeds back to worsen insulin resistance, creating a vicious cycle of perpetuation [22]. High insulin levels are also responsible for central adiposity, a phenomenon that is more prevalent in women with PCOS compared to non-PCO controls [23]. Adipose tissue is recognized for serving as a reservoir as well as site of metabolism for androgens [24].

Anti-mullerian hormone (AMH), an important marker of ovarian reserve, and vascular endothelial growth factor (VEGF), a crucial factor in angiogenesis, have in recent years received attention as plausible players of causative relevance for PCOS. Circulating levels of AMH are significantly higher in women with PCOS at all ages; AMH levels have been correlated with circulating androgens as well as with insulin resistance, and a role for AMH in PCOS pathogenesis is hypothesized [25,26,27]. Given that elevated AMH levels are near ubiquitous in PCOS populations, propositions have been attempted to incorporate AMH levels as a possible diagnostic tool for PCOS [28]. Unlike AMH, VEGF is better understood for its role in ovarian angiogenesis than in the pathogenesis of PCOS [29].

## 3. Vitamin D and PCOS

In this review we summarize our current understanding of the relevance of vitamin D signalling for mechanisms that are known to be relevant to the pathophysiology of PCOS.

A literature review was performed using PubMed, Google Scholar, ClinicalKey, Academia, Cochrane Database and Mendeley. Specific MeSH words including vitamin D, polycystic ovarian syndrome, insulin-resistance, anti-mullerian hormone and vascular endothelial growth factor were used to identify relevant studies. Two independent researchers (R.K, L.P) selected relevant articles in a two-step process. First step included reviewing abstracts for potential eligibility and relevance. This was followed by retrieval of full texts for detailed evaluation. All articles published in the English language were considered for inclusion and focus on clinical relevance was prioritized; non-English publications were excluded.

### 3.1. Vitamin D—A Hormone

A sex-steroid hormone that is universally known for its relevance for skeletal health, vitamin D has been a focus of much attention in the field of PCOS in recent years. There is undisputed evidence of the essential role that vitamin D plays in bone metabolism and in the maintenance of overall skeletal health [30]. In the past decade vitamin D has gained increasing attention for its myriad extra skeletal effects and biological responses including anticarcinogenic effects, association with cardiovascular health, protection against certain chronic and autoimmune illnesses [30,31,32]. Furthermore, there is growing evidence supporting a pivotal role of vitamin D in reproductive health [33,34,35,36]. Serum level of less than 20 ng/mL is commonly utilized to reflect vitamin D deficiency, levels between 20 ng/mL to <30 ng/mL reflect insufficiency whereas ≥30 ng/mL are commonly recognized to represent normal vitamin D status [37].

### 3.2. Vitamin D—Relevance in PCOS?

Vitamin D is recognized to play a crucial role in regulating the expression of genes involved in glucose and lipid metabolism [38]. Observational data as well as experimental studies provide convincing evidence relating vitamin D deficiency to many of the endocrine, metabolic and clinical hallmarks of PCOS. Vitamin D deficiency and insufficiency have been associated with many of the overt and covert phenomenon that are prevalent in PCOS including ovulatory dysfunction [34], hyperandrogenemia [39], insulin resistance [40], indices of adiposity [41], risk for diabetes [42] dyslipidaemic and systemic proinflammatory milieus [43]. An overview by Colonese et al. provides a comprehensive review of gynaecological and obstetrical outcomes that have been related to vitamin D signalling [44]. In this section, we review data that relate vitamin D to distinct biological pathways that have been implicated in the pathophysiology of PCOS.

### 3.3. Vitamin D—Bio-Activation and Mechanism of Action

**Figure 1:**
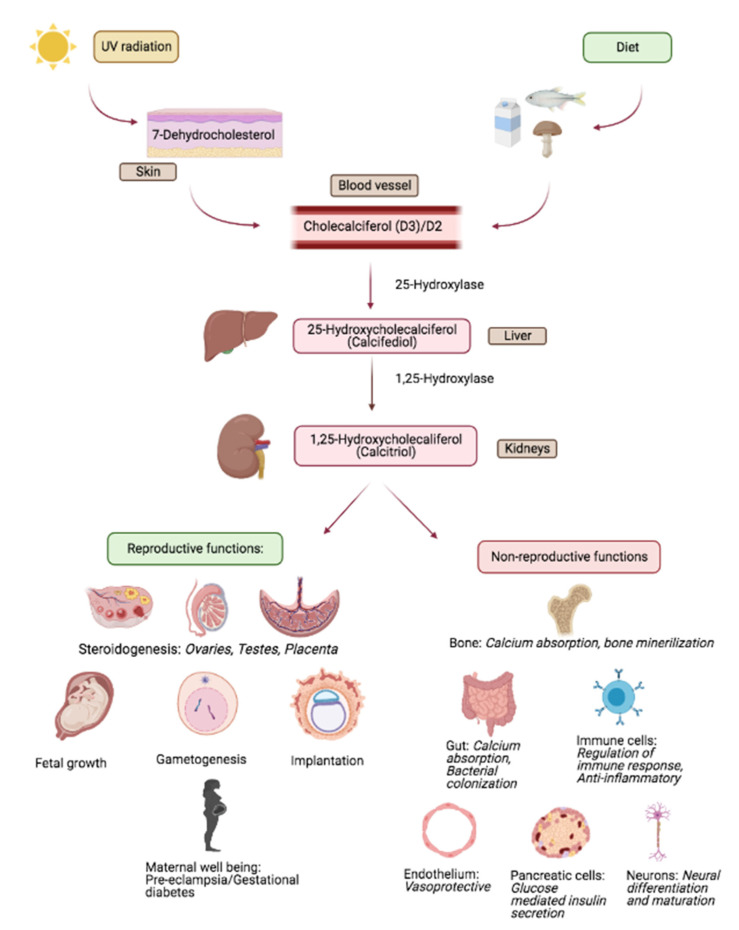
Overview of vitamin D metabolism and salient reproductive and non-reproductive actions. Adapted from study by Luk et al. [52].

Vitamin D is a fat-soluble vitamin that acts as a steroid hormone. Figure 1 provides a simplified schema of the bioavailability, metabolism and target effects of vitamin D; for in depth review, please see reference [45] and citations therein [45]. Briefly, the primary source of vitamin D for humans include sunlight, diet and dietary supplements. Vitamin D has two major forms; D2 (ergocalciferol) and D3 (cholecalciferol) [46]. Vitamin D2 is mainly derived from plants and is synthesized from ergosterol; yeast and sun dried/UV mushrooms are rich sources of D2. Vitamin D3 is innately of animal origin, including being endogenously synthesized in humans. On exposure to sunlight, solar ultraviolet B radiation acts on the skin and converts 7-dehydrocholesterol to pre-vitamin D3 which is immediately converted to vitamin D3. Dietary vitamin D (D2 as well as D3) gets transported into the circulation via lymphatics by getting incorporated into chylomicrons. 

Bio-activation of vitamin D occurs in a two-step process. First, in the liver, the enzyme 25 hydroxylase metabolizes vitamin D (D2/D3) into 25-hydroxyvitamin D (25(OH)D); circulating levels of 25(OH)D represent the overall vitamin D status, despite the fact that this represents the inactive form of the vitamin; as per the Endocrine Society, serum levels of 25(OH)D at <30 ng/mL reflect evidence of vitamin D deficiency [47]. Activation of 25(OH)D occurs primarily in the renal proximal convoluted renal tubules mediated by the enzyme 1-alpha (α) hydroxylase that catalyses the conversion of 25(OH)D into 1,25 dihydroxy-vitamin D (1,25(OH)2D) which is the metabolically active form of vitamin D that mediates its actions through binding to the cognate vitamin D receptor (VDR). More recently, 1-alpha (α) hydroxylase has been identified in non-renal tissues suggesting that many target tissues and cells retain an ability to focally activate 25(OH)D to 1,25(OH)2D [48,49]. Interaction of 1,25(OH)2D with VDR initiates a cascade of events that involves additional co-regulatory proteins to eventually bring about transcription of vitamin D response genes [50]. Non-genomic mechanisms of vitamin D action are also recognized [51].

### 3.4. Vitamin D Receptor (VDR)

**Figure 2:**
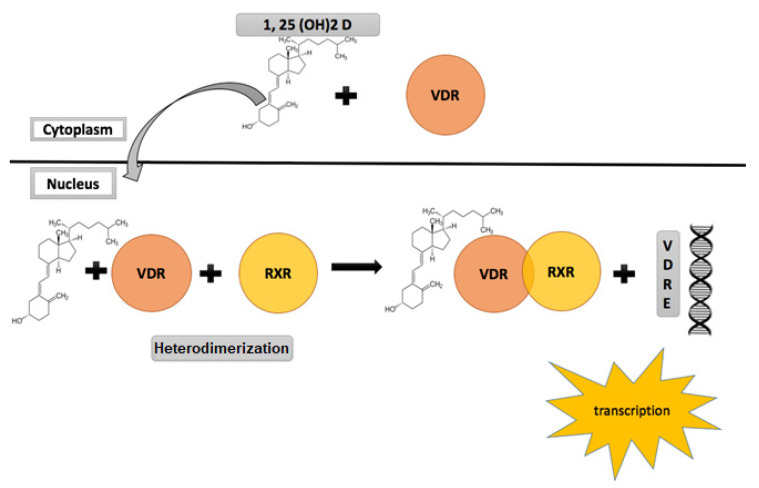
Vitamin D receptor (VDR) signalling and activation.

Belonging to the nuclear receptor superfamily, the nuclear VDR in response to ligand binding, and through mediation of co-activators, acts as a transcription factor and serves as the gateway for all genomic actions of vitamin D. It is expressed in multiple human tissues including the skeleton, intestines, renal tissue parathyroid glands [53]. Animal studies have shown that apart from these calcium regulating organs, VDR is also widely expressed in higher centres and organs of reproduction including the hypothalamus, pituitary, ovaries, uterus, placenta and the testes [53,54,55]. These findings have led to an increasing curiosity about the role of vitamin D in the physiology and pathology of reproduction [34]. 1α,25-(OH)_2_ D is the active ligand for VDR. A heterodimer complex is formed between VDR and the retinoic acid receptor and this heterodimer-initiated signalling mediates the various biological actions of the active form of vitamin D [56] (Figure 2). Non-genomic actions of vitamin D while recognized and understood to be mediated via a membrane located VDR are relatively less understood. For an in-depth review of vitamin D signalling, please see references [50,51], and citations therein.

### 3.5. Vitamin D, AMH and PCOS

Ovarian follicular reserve is defined as the reproductive potential at any reproductive age based on the quantity and quality of available residual oocytes at any given age and is commonly utilized as an indicator for female fecundity [57]. Serum levels of follicle stimulating hormone (FSH), estradiol and Inhibin B (in the early follicular phase of menstrual cycle) are amongst the earliest recognized markers of ovarian reserve. In recent years, ultrasound based ovarian antral follicle count (AFC) and serum levels of AMH have emerged as robust reflectors of residual ovarian reserve; either of these can provide reliable estimates of ovarian reserve without being dependent on timing of assessment in relation to phase of menstrual cycle. AMH, also known as the mullerian inhibiting substance (MIS) is a homodimeric glycoprotein that belongs to the family of transforming growth factors (TGF-β) [58]. AMH levels are almost absent during infancy, increase during puberty indicating a rise in primordial follicle recruitment and eventually fall to undetectable levels by menopause [59,60,61]. It is produced exclusively by the granulosa cells of the ovary where a higher expression of AMH and AMH receptor mRNA are found [60,61,62,63]. AMH is considered one of the best markers of ovarian reserve as the serum levels do not fluctuate significantly during the menstrual cycle [60,61,63]. However, its overall levels can be affected by biological and extraneous influences, such as obesity, use of hormonal contraceptives and vitamin D deficiency [64,65,66].

**Figure 3:**
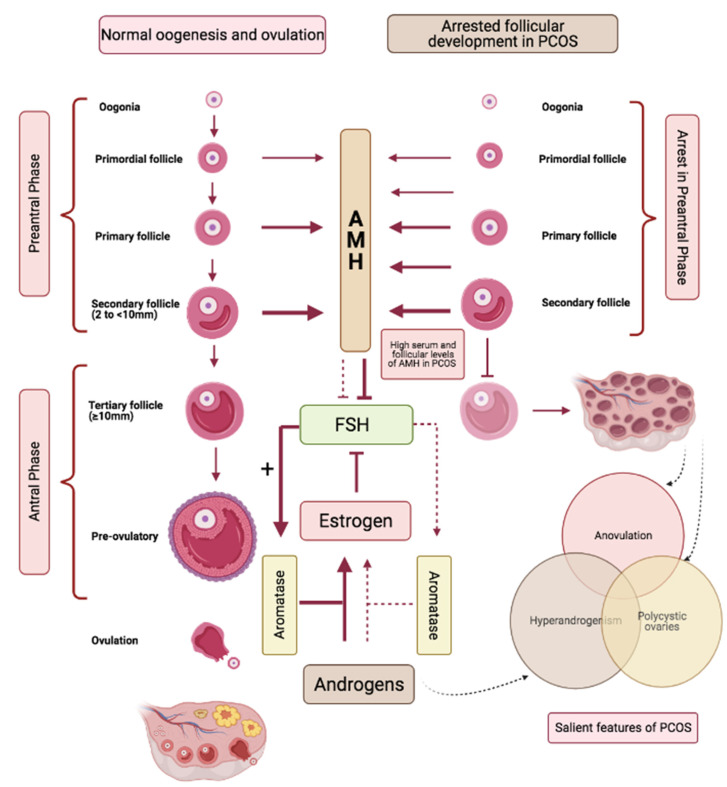
Parallel pathways demonstrating the role of Anti-mullerian hormone (AMH) in follicular development in normal (**left**) versus polycystic (**right**) ovaries. AMH is produced by the growing follicles and production ceases when the follicles reach a size ≥10 mm. AMH keeps FSH signalling in check. High levels of AMH in PCOS cause increased suppression of FSH signalling and result in (i) arrest in follicular growth, (ii) reduced activation of FSH mediated aromatase activation that contribute to increased androgen levels.

AMH is secreted by the pre-antral and small antral follicles measuring ≤4 mm and ceases when follicle size reaches ≥10 mm (Figure 3) [67,68]. In PCOS, the polycystic ovarian morphology is a sine qua non of high antral follicle repertoire, and aligned with this, serum levels of AMH are higher in women with PCOS compared to those without polycystic ovaries [69]. Additionally, follicular fluid levels of AMH are also found to be significantly elevated in women with PCOS [70]. Figure 3 provides a simplified schema identifying a role for AMH in controlling the ovarian follicular dynamics in response to FSH signalling, as well as outlining a disarrayed paradigm in PCOS. In simple terms, AMH keeps FSH signalling in check; a lowering of AMH, as occurs with advancing age, contributes to exaggerated FSH signalling that can manifest as spontaneous multi-follicular ovarian response commonly observed in women of advanced reproductive age, and can explain the predisposition of aging reproductive women to spontaneous twin conceptions [71]. Conversely, high AMH levels, as are hallmarks of polycystic ovarian phenotype, dampen FSH signalling, resulting in the arrested follicular growth and anovulation that exemplify the ovarian dynamics of PCOS. The ovarian enzyme aromatase is under direct control of FSH and is responsible for the conversion of ovarian androgens to estrogens; high AMH in PCOS, by suppressing FSH signalling thus not only is causative to arrested follicular development, but by suppressing conversion of ovarian androgens to estrogens, is causative to for both focal and systemic androgen excess of PCOS (Figure 3) [25].

**Figure 4:**
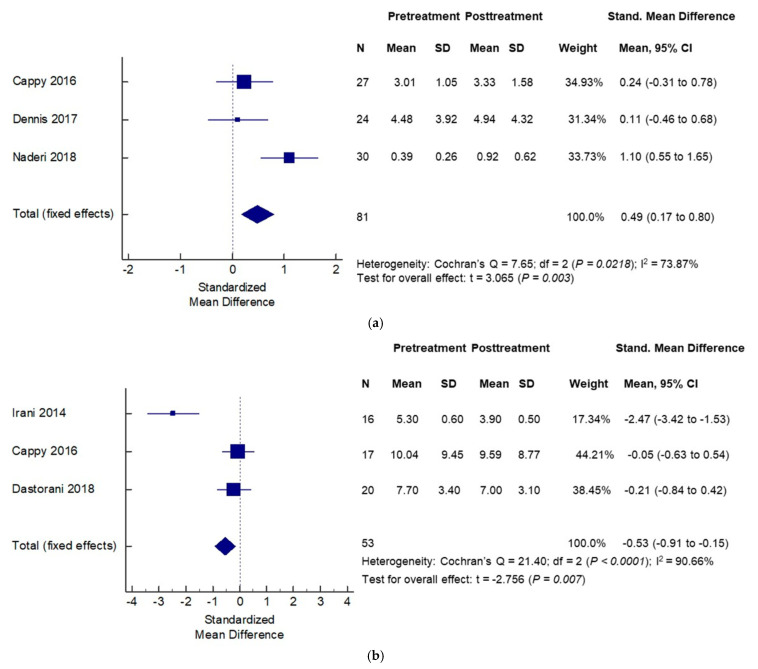
Effects of vitamin D supplementation on serum anti-mullerian hormone (AMH) levels differ by PCOS status: —effects seen in non-PCOS (**a**) and PCOS (**b**) populations. Adapted from Mordi et al. [73].

Vitamin D appears to differentially impact not just circulating levels of AMH, but also the intersection of AMH and ovarian follicular dynamics in women with and without PCOS. Irani et al. showed that in vitamin D deficient women with PCOS, supplementation with vitamin D caused a significant decrease in the abnormally elevated AMH levels in this population [72]. A recent systematic review of 18 observational and 6 interventional studies examined the impact of vitamin D supplementation on AMH levels in women with PCOS. Authors reported a complex cause-effect relationship such that the direction of causality depended on the ovulatory status of the population. Vitamin D supplementation was followed by a decrease in AMH levels in patients with an-ovulatory PCOS; in contrast, vitamin D supplementation in the ovulatory PCOS population was followed by an increase in AMH levels (Figure 4a,b) [73]. A possible explanation for the observed effects of vitamin D supplementation on circulating AMH levels may lie in the presence of a vitamin D response element on AMH gene promoter region [74,75]. Could such an effect of vitamin D supplementation on lowering of AMH levels in anovulatory women with PCOS, as reported by Irani et al., be harnessed to improve ovulatory response in women with PCOS? Indeed, facilitatory modulation of ovarian follicular dynamics with vitamin D supplementation was suggested in women with PCOS in a prospective, double blind placebo-controlled trial, when addition of vitamin D and calcium to metformin resulted in improved attainment of spontaneous menses and attainment of dominant follicle compared to other groups (metformin alone, vitamin D plus calcium and placebo) [76].

### 3.6. Vitamin D, Androgens and PCOS

The adrenal glands, the ovarian theca and peripheral tissues serve as sources of circulating androgens in females. Hyperandrogenemia in PCOS is primarily of ovarian origin, although some level of adrenal contributions to excess androgens are not uncommon. In classic PCOS, androgen excess can begin as early as puberty, especially in the setting of premature adrenarche and precocious puberty. Manifestations of hyperandrogenemia can range from no symptoms to classic symptoms of androgen excess (acne, hirsutism and alopecia) to menstrual and ovulatory dysfunction. Symptoms of hyperandrogenesim can not only take a toll on the physical and psychological health of women but are also associated with significant pregnancy complications including preterm delivery and pre-eclampsia [77].

Sex hormone binding globulin (SHBG) is an important carrier protein that regulates free androgen levels [78]. SHBG binds to circulating testosterone and androstenedione, thereby minimizing the percentage of available free androgens to act on target tissue. States of insulin resistance including PCOS are found to be associated with lower SHBG levels [79]. Insulin resistance and hyperinsulinemia contribute to hyperandrogenemia through reducing hepatic production of SHBG, as well as by direct stimulant effects of excess insulin on the ovarian production of androgens by the ovarian theca [80]. Vitamin D can modulate circulating androgens through multiple pathways. A positive correlation between serum vitamin D levels and SHBG levels have been shown [81]. In a pilot study of vitamin D supplementation undertaken in an overweight to obese population of women with PCOS, significant lowering in circulating androgens (total testosterone, androstenedione and DHEAS) were observed following 3 months of supplementation with high dose vitamin D and calcium [82]. These findings were corroborated in the recent review article that included and analysed 9 different RCTs which showed that high dose supplementation of vitamin D (4000 IU) compared with low dose (1000 IU) and placebo were associated with beneficial effects not only on free testosterone but also SHBG and free androgen index (FAI) [83]. Data from in vitro experiments utilizing human adrenocortical cell line provide convincing evidence of suppressive effects of vitamin D on steroidogenic enzymes with resulting lowering of levels of steroid intermediaries including androgens in the culture medium following treatment with (1,25(OH)2D3) [84].

### 3.7. Vitamin D, Metabolic Dysfunction and PCOS

**Figure 5:**
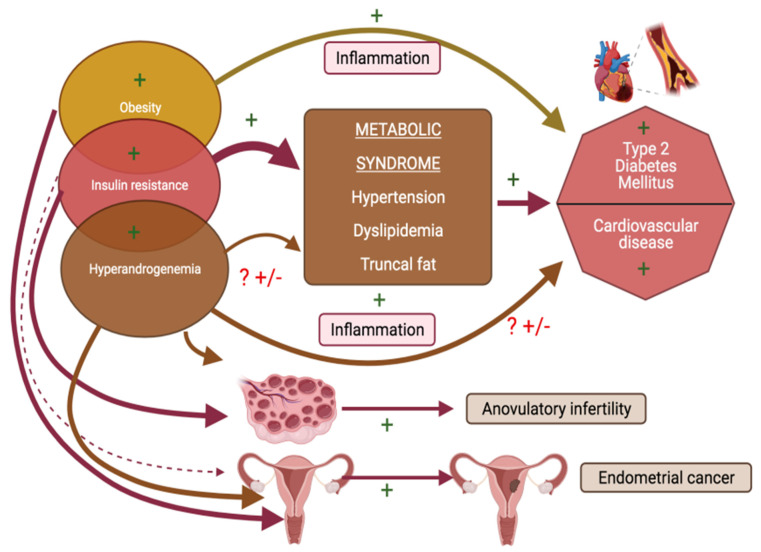
Schema outlining pathophysiology of polycystic ovarian syndrome (PCOS) related health burdens and targets for facilitatory effects of vitamin D (indicated by +).

Woman with PCOS are more prone to metabolic derangements including insulin resistance (IR), dyslipidaemia, hypertension, obstructive sleep apnoea and hence eventually are at an increased risk of developing cardiovascular diseases in the long term (Figure 5) [85]. Patients with type 2 diabetes mellitus are also at an exaggerated risk for vitamin D deficiency [86]. A relevance of vitamin D signalling for glucose homeostasis is well recognized and vitamin D is considered to potentially exert its effects on glucose metabolism via genomic and non-genomic pathways, and through direct as well as indirect effects (latter mediated via intermediary effects on processes of inflammation). Vitamin D signalling via VDR, enhances the genomic stimulation of insulin receptor mRNA [87,88]. This in turn activates insulin synthesis and release, as well as also inhibits certain pro-inflammatory cytokines which are recognized players in the pathogenesis of IR [89].

Limited data suggest that addition of vitamin D to insulin sensitizer regimens such as metformin and inositol isomers may offer benefit in PCOS [90]. Advani et al. showed that 12-week supplementation with insulin sensitizing agents (the inositol isomers myo-inositol (MI) and D-Dhiro-inositol (DCI) and chromium picolinate), plus antioxidants (N-acetyl cysteine and lycopene) and vitamins (including vitamin D, biotin and folic acid) was associated with regular menstrual cycles, improved hirsutism and significant reduction the BMI in obese patients with PCOS compared to baseline [91]. Despite the benefits achieved in this latter study, it is difficult to tease out the contributions of individual components of the supplemental cocktail utilized and additional studies are needed to examine if addition of vitamin D to inositol isomers offers any additive benefit in the PCOS population.

With regards to lipid metabolism, vitamin D can cause stimulation of liver microsomal triglyceride transfer protein (MTP) by increasing intracellular calcium levels. MTP is a dimeric protein involved in lipid transport (triglycerides, cholesteryl ester, phospholipid) across membranes. MTP participates in formation and subsequent secretion of VDRL, which in turn reduces the circulating level of total serum cholesterol [92].

A number of meta-analysis have aimed to determine the effect of vitamin D supplementation on the different metabolic biomarkers in women with PCOS as shown in Table 2. As is evident, the existing data are limited by heterogeneity and small sample sizes of studied populations, heterogeneity in study designs and interventions as many of included co-supplementation of vitamin D with metformin, oral contraceptive pills, omega-3- fatty acids, probiotics or other nutrients, thus limiting our ability to tease out individual effects of vitamin D. A recent meta-analysis by Guo et al. however included thirteen RCT trials (824 patients) which focused on effect vitamin D supplementation alone on various metabolic parameters of PCOS [93]. The authors showed that sole supplementation of vitamin D was associated with significant lowering of fasting plasma glucose (FPG) levels [Standardized mean difference (SMD): −0.34, 95% CI: −0.61, −0.07]. Heterogeneity between studies was found to be high, one of the contributors being type of vitamin D supplemented. This was eliminated by removing a study that used calcitriol [94]. The beneficial effect on FPG was found to be stronger by taking intake manner into account (daily versus weekly) and this association was found to be independent of the baseline vitamin D deficiency among these patients. They also showed that vitamin D supplementation compared to placebo resulted in significant improvements in indices of insulin resistance including decrease in fasting insulin levels (SMD: −0.43, 95% CI: −0.67, −0.18), HOMA-IR (SMD: −0.25, 95% CI: −0.47, −0.02) and increase in QUICKI (SMD: 0.52, 95% CI: 0.11, 0.92). Furthermore, the analysis included trials assessing effect of vitamin D supplementation on lipids, where they reported a significant lowering in serum VLDL-C levels (SMD: −0.18, 95% CI: −0.44, 0.09) but no effect on LDL-C (SMD: −0.23, 95% CI: −0.60,0.14), HDL-C (SMD: 0.15, 95% CI: −0.03, 0.33) and on triglycerides (SMD: −0.23, 95% CI: −0.50, 0.03).

### 3.8. Vitamin D, VEGF and Ovarian Angiogenesis in PCOS

There is a growing interest in the area of ovarian angiogenesis and its role in pathophysiology of PCOS [95]. Angiogenesis is a process of new blood vessel formation. In adults, the physiological process of angiogenesis is mainly found in wound healing and reproductive tissues as rest of the vasculature remains quiescent. The role of angiogenesis in the development of cancer and cardiovascular disease is well understood. Every menstrual cycle is regulated by a precise balance between formation and regression of blood vessels [96]. This balance plays a vital role in follicular development, maturation, oocyte quality, ovulation, corpus luteal regression and thus in fertility. Various angiogenic factors and associated proteins including vascular growth factor (VEGF), placental growth factor (PIGF), transforming growth factor beta1 (TGFβ1) and basic fibroblast growth factor (bFGF) play a key role in establishing this balance [97]. At the same time, antiangiogenic factors like thrombospondins (TSP), endostatins, angiostatins and soluble FLT 1 act to oppose excess angiogenesis and hence formation of tortuous vessels [90]. Of these factors, VEGF, a heparin binding heterodimer is found to be the most important regulator of angiogenesis. It exists in 6 isoforms: VEGF A, B, C, D, E and PGIF. VEGF binds to VEGFR2/R1/kinase insert domain receptor (KDR), expressed in ovarian cells including granulosa and theca cells [98]. It exerts its action by promoting endothelial cell proliferation, migration and vascular permeability. PIGF is a VEGF family member dimerizes with VEGF and supports vessel growth [99].

Battaglia et al. published the first article describing a possible role of ovarian vascularization in PCOS pathology and diagnosis. This was a case control study where they used Doppler USN to measure ovarian volume, echo-density and follicular number in subjects with and without PCOS. Interestingly they found an elevated pulsatility index (PI) and a decrease in resistance index (RI) showing a higher incidence of ovarian neoangiogenesis in PCOS cases over controls [100] (Battaglia et al.). Since then, multiple studies have shown that patients with PCOS have increased VEGF levels in both serum and follicular fluid with an increase in ovarian stromal vascularization [101,102,103].

**Figure 6:**
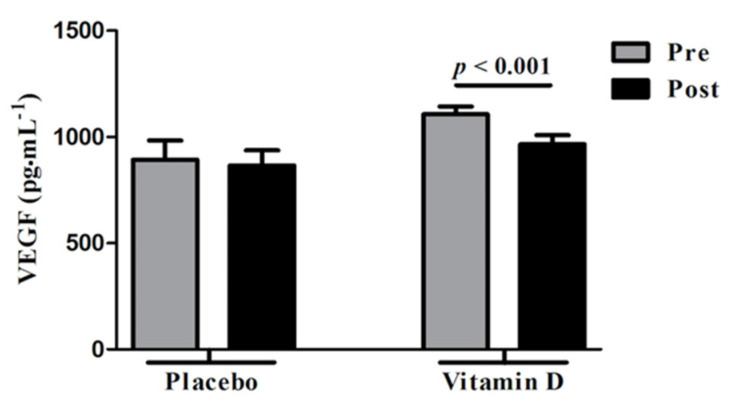
Effect of vitamin D supplementation on vascular endothelial factor (VEGF) levels in comparison with placebo. Vit D supplementation was associated with significantly reduced serum VEGF levels in vitamin D deficient women with polycystic ovarian syndrome (PCOS). No significant changes were observed with placebo. Pre: Before vitamin D or placebo, Post: After vitamin D or placebo administration. Reprinted/Adopted from reference [110], with permission.

Ovarian hyperstimulation syndrome (OHSS) is a major and potentially fatal complication of controlled ovarian stimulation during assisted reproductive technologies (ART) [104] and VEGF has emerged as a major player in the pathogenesis of OHSS [104,105,106,107]. Since the main underlying pathophysiology for development of OHSS include increased vascular permeability following exposure to human chorionic gonadotropin (hCG) that is commonly used to trigger oocyte maturation prior to egg retrieval in ART, strategies to prevent OHSS aim at minimizing robustness of follicular response in ART cycles with use of lower gonadotropin doses, by reducing exposure to hCG through dose reduction (not an effective approach) or most effectively by substituting GnRH agonist for hCG; GnRH agonist induces an endogenous surge of LH (LH has a much shorter half-life than hCG) with a lesser magnitude of impact on VEGF overexpression compared to hCG [104]. Vitamin D has shown to decrease VEGF production in both human cancer cells [108] and in animal studies [109]. In a randomized placebo-controlled trial that examined effects of vitamin D versus placebo administration in vitamin D deficient women with PCOS, Irani et al. [110] demonstrated significant reduction in serum VEGF levels in the group supplemented with vitamin D compared to placebo (1106.4 ± 36.5 to 965.3 ± 42.7 pg·mL^−1^; *p* < 0.001) (Figure 6).

### 3.9. Vitamin D and PCOS—Teasing out of Associations from Causative Relationships

It is through the data emanating from studies of vitamin D supplementation that a causative relevance of vitamin D insufficiency for biochemical underpinnings to and hallmarks of PCOS is emerging [111,112,113,114,115], (Table 2—Further explanation under section on metabolic dysfunction and PCOS).

## 4. Summary

This review summarizes our current understanding of the mechanisms relevant to the pathophysiology of PCOS and examines the role of vitamin D signaling in this context. Considering the existing and reviewed data and given a recognized safety profile, vitamin D supplementation may be judiciously considered as a possible safe cost-effective intervention that aims at mitigating biochemical and clinical stigmata and as a risk attenuation strategy (for OHSS) in patients diagnosed with PCOS.

## Figures and Tables

**Table 1:** Diagnostic Criteria of Polycystic Ovary Syndrome.

Year	Institute	Consensus Criteria
1990	NICHD/NIH	Patient demonstrates both: Clinical and/or biochemical hyperandrogenism, and Oligo-ovulation or chronic anovulation
2003	ESHRE/ASRM (Rotterdam)	Patient demonstrates at least two of three criteria: Oligo-or chronic anovulation Clinical and/or biochemical hyperandrogenism Polycystic ovarian morphology
2006	AES	Patient demonstrates both: Clinical hyperandrogenism and/or biochemical hyperandrogenism, and Oligo-anovulation and/or polycystic ovaries

NIH/NICH: National Institute of Health/National Institute of Child Health and Human Disease ESHRE/ASRM: European Society of Human Reproduction and Embryology/American Society for Reproductive Medicine; AES: Androgen Excess Society.

**Table 2:** RCT evidence on the effects of vitamin D supplementation on clinical and metabolic parameters pertinent to PCOS.

Study Design/Reference	Sample Size	Population	Intervention	Duration	Regimen	Study Parameters	Conclusions
Randomized, double blinded, placebo controlled trial Dastorani et al. [111]	N = 40 (1:1 randomization)	40 infertile PCOS patients who were IVF candidates PCOS diagnosis based on Rotterdam criteria Vitamin D deficiency not diagnosed at the beginning of the study	Either vitamin D or placebo (paraffin capsules)	8 weeks	50,000 IU vitamin D or placebo every other week	AMH, insulin, HOMA-IR, insulin sensitivity check index (QUICKI), serum and total cholesterol	Compared to placebo, vitamin D supplementation resulted in significant lowering of serum AMH, insulin levels, HOMA-IR, serum total and LDL cholesterol level and improved QUICKI
Randomized, double blinded, placebo controlled trial Javed et al. [112]	N = 40 (1:1 randomization)	40 PCOS patients who were vitamin D deficient PCOS diagnosis based on the Rotterdam criteria	Either vitamin D (n = 20) or placebo (n = 20)	12 weeks	3200 IU vitamin D or placebo daily	hs-CRP, lipid profile, insulin, HOMA-IR, glucose, Weight, BMI, FAI, Testosterone, SHBG, ALT, HA, PIIINP, TIMP-1, ELF score	Compared to placebo, vitamin D resulted in modest improvement in insulin sensitivity indices, significant reduction in ALT; there was no effect of vitamin D supplementation on CVD risk markers and hormones
Randomized, double blinded, placebo controlled trial Ostadmohammadi et al. [113]	N = 60 (1:1 randomization)	60 PCOS patients PCOS diagnosis based on Rotterdam criteria Vitamin D deficiency not diagnosed at the beginning of the study	Vit D + probiotic vs. placebos (corn oil and starch)	12 weeks	50,000 IU vit D + 8 × 10^9^ CFU/day probiotic or placebos every 2 weeks	Serum TT, hs-CRP, SHBG, NO, TAC, GSH, MDA, hirsutism (mFG scoring), mental health (BDI, GHQ-28, DASS), quality of sleep (PSQI)	Compared to placebo, vitamin D supplementation resulted in decrease in serum total testosterone level, improved hirsutism, lowering of hs-CRP, plasma TAC, GSH and MDA levels and had positive effects on mental health parameters compared to placebo. No significant effect was observed on SHBG, PSQI, plasma NO, acne and alopecia
Randomized, double blinded, placebo controlled trial Jamilian et al. [114]	N = 60 (1:1 randomization)	60 PCOS patients PCOS diagnosis based on Rotterdam criteria Vitamin D deficiency not diagnosed at the beginning of the study	Vit D + omega 3FA vs. placebo	12 weeks	50,000 IU vitamin D every 2 weeks + 2000 mg omega 3 fatty acid/day OR placebo every 2 weeks	Total testosterone, SHBG, FAI, GSH, CRP, MDA, NO, TAC, IL-1, VEGF, hirsutism (mFG), depression and anxiety (BDI, DASS, GHQ-28)	Compared to placebo, vitamin D + omega 3 FA co-supplementation resulted in decrease in serum total cholesterol, hs-CRP, MDA, caused down regulation of IL-1, VEGF, increased TAC and showed improvement in BDI, DASS scores. No significant difference were observed in SHBG, FAI, plasma NO levels and GSH
Randomized, Double blinded, Placebo controlled trial Trummer et al. [115]	N = 123 (2:1 randomization)	123 Patients who were vitamin D insufficient PCOS diagnosis based on Rotterdam criteria Vitamin D insufficiency diagnosed as <75 nmol/L (<30 ng/mL) per the Endocrine society [47]	Vitamin D (50 oily drops with cholecalciferol) (81) vs. placebo (similar oily drops without cholecalciferol) (41)	24 weeks	20,000 IU vitamin D3 weekly OR placebo weekly	AUC gluc during OGTT, HOMA-IR, total cholesterol, HbA1C, TT, FT, menstrual frequency, insulin sensitivity (QUICKI), TG	Compared to placebo, vitamin D resulted in decrease in plasma glucose during 60 min OGTT following 75 g glucose load ingestion at 12- and 24-week visit. No significant effect was observed on AUG gluc at end of 24 weeks and on other metabolic/endocrine parameters

AMH: Anti-mullerian hormone; TT: Total testosterone; FT: Free testosterone; PIINP: N-terminal pro-peptide of type III pro-collagen; TIMP-1: Tissue inhibitor of metallo-prteinases-1; ELF: Enhanced liver fibrosis; HA: Hyaluronic acid; mF-G: Modified Ferriman-Gallewey scoring system; BDI: Beck depression inventory; GHQ-28: General health questionnaire-28; DASS: Depression anxiety and stress scale; PSQI: Pittsburgh sleep quality index; NO: Nitrous oxide; hs-CRP: high-sensitivity C-reactive protein; GSH: Total glutathione; TAC: Total anti-oxidant capacity; MDA: Malondialdehyde; AUG gluc: Plasma glucose area under curve; HOMA-IR: Homeostatic model assessment-insulin resistance; QUICKI: Quantitative insulin sensitivity check index; TG: Triglycerides.
